# Factors associated with death or intensive care unit admission due to pandemic 2009 influenza A (H1N1) infection

**DOI:** 10.4103/1817-1737.78429

**Published:** 2011

**Authors:** Payam Tabarsi, Ahmadreza Moradi, Majid Marjani, Parvaneh Baghaei, Seyed Mohammadreza Hashemian Hashemian, Seyed Alireza Nadji, Atefeh Fakharian, Davood Mansouri, Mohammadreza Masjedi, Aliakbar Velayati

**Affiliations:** 1 Mycobacteriology Research Center Virology Research Center, NRITLD, Masih-Daneshvari Hospital, Shahid Beheshti University of Medical Sciences, Tehran, Iran; 2 Nursing and Respiratory Health Management Research Center, NRITLD, Masih-Daneshvari Hospital, Shahid Beheshti University of Medical Sciences, Tehran, Iran; 3 Clinical Tuberculosis and Epidemiology Research Center, NRITLD, Masih-Daneshvari Hospital, Shahid Beheshti University of Medical Sciences, Tehran, Iran; 4 Chronic Respiratory Disease Research Center, NRITLD, Masih-Daneshvari Hospital, Shahid Beheshti University of Medical Sciences, Tehran, Iran; 5 Pediatric Respiratory Disease Research Center, NRITLD, Masih-Daneshvari Hospital, Shahid Beheshti University of Medical Sciences, Tehran, Iran

**Keywords:** Hospital mortality, influenza a virus H1N1 subtype, intensive care units, risk factors

## Abstract

**BACKGROUND:**

In preparation for pandemic HINI or H1N1 influenza (H1N1) it is necessary to identify factors associated with mortality of patients with HINI and hospital admissions to intensive care unit (ICU) of patients diagnosed in 2009 with HINI.

**OBJECTIVES:**

To describe the clinical and epidemiological features associated with 2009 HIN1 mortality and ICU patient admissions to Masih Daneshvari Teaching Hospital, Iran.

**METHODS:**

A retrospective cross-sectional study was conducted among patients with mortality and admissions to ICU with confirmed HINI. Demographic, clinical, laboratory, radiological findings, and epidemiologic data were abstracted from medical records, using a standardized datasheet.

**RESULTS:**

From June through December 2009, 20 out of the 46 confirmed hospitalized patients with confirmed H1NI were admitted to the ICU and 7 (15%) died. Among various variables, opium inhalation (*P* = 0.01), having productive cough, hemoptysis, chest pain, confusion, and loss of consciousness were significantly related to ICU admission (*P* < 0.05). Pleural effusion (*P* = 0.006), elevated liver enzymes, as well as CPK and LDH level were significantly relevant to ICU admission (*P* < 0.05). Delayed antiviral treatment was more common among patients who died and the elderly.

**DISCUSSION:**

Patients who were admitted to ICU with confirmed H1N1 included the following risk factors: delayed initiation of antiviral therapy, history of opium inhalation and symptoms including; productive cough, hemoptysis, chest pain, confusion, and loss of consciousness. The mortality rate in the study population was high but compares favorably with other recent published studies.

On 11 June 2009, the World Health Organization formally confirmed the first pandemic of influenza for 40 years.[1] A novel influenza A (H1N1) virus, which is a genetic resentment of four influenza A viruses (i.e., swine influenza, human seasonal influenza, avian influenza, and Eurasian swine), began to cause illness in Iran three months after it first emerged in Mexico in March 2009.[2–4]

In the context of 2009 pandemic, clinicians were uncertain regarding the clinical and laboratory findings of H1N1 pneumonias (H1N1). Therefore, identifying factors associated with the death or intensive care unit (ICU) admission of hospitalized patients with 2009 H1N1 is critical in preparation for the potential waves of pandemic H1N1 influenza. Current reports have suggested in addition to many of the previously known risk factors for complications of seasonal influenza, underlying co-morbidities and delayed initiation of antiviral therapy as risk factors for severe disease.[5–8]

Although during the 2009 pandemic outbreak a large number of individuals who referred to the Emergency Department (ED) with influenza-like illnesses (ILIs) were admitted to regular wards, few patients were admitted to the ICU.

## Objective

To describe the clinical and epidemiological features associated with ICU admission and death of hospitalized patients with 2009 H1N1.

## Methods

A retrospective cross-sectional study was conducted among patients who were hospitalized with confirmed 2009 H1N1 virus infection. Demographic, clinical, laboratory data included; complete blood count, liver function test, erythrocyte sedimentation rate (ESR), creatinine blood level (Cr), creatine phosphokinase blood level (CPK), lactate dehydrogenase blood level (LDH), O_2_ saturation, sputum culture for bacteria and blood culture, radiological findings, and epidemiologic data were extracted from medical records, using a standardized datasheet. The comparison was done between ICU admitted patients and non-ICU patients. Clinical and laboratory data were compared between patients who died and those who survived.

### Setting

Masih Daneshvari Teaching Hospital is the largest tertiary health care center for patients with respiratory diseases in Tehran, Iran. During the outbreak of 2009 pandemic, this hospital was a reference center for H1N1 cases in Tehran, with the aim of controlling the pandemic. The study was conducted during June through December 2009. All admitted patients with laboratory confirmed H1N1 influenza were included in the study.

### Laboratory confirmation

Respiratory tract specimens (including; nasopharyngeal aspirates/swabs, and endotracheal/bronchoscopic aspirates) were properly treated at the virology laboratory immediately. Nucleic acid was extracted with QIAamp^®^ Viral RNA Mini Kit (QIAGEN GmbH, Germany) and Invisorb^®^ Spin Virus RNA Mini Kit (Invitek, Germany). cDNA was synthesized by RevertAid^TM^ H Minus First Strand cDNA synthesis kit (Fermentas LIFE SCIENCES) according to the manufacturer’s instruction. The presence of the pandemic H1N1 2009 infection was confirmed by real-time reverse-transcriptase-polymerase-chain-reaction (rRT-PCR), run on BioRad CFX96^TM^ real time PCR machine (USA), according to the protocol developed by the Center for Disease Control (CDC), USA.[9]

### Statistical analysis

Statistical analyses were performed using the SPSS software. The two-sided χ^2^ test was used for comparison of categorical variables, with Fisher’s correction when needed. The *t*-test was used for comparison of the continuous variables. To analyze for the ICU admission and mortality, we used logistic regression. A two-tailed *P*-value of less than 0.05 was considered statistically significant. To determine the independent risk factors, a multivariate logistic regression analysis was performed for the factors which had *P* < 0.2 in univariate analysis.

### Ethics

The study was approved by the institutional ethics committee.

## Results

From June through December 2009, 125 of 656 patients who were referred to the hospital with influenza-like illnesses (ILIs) were admitted to regular wards. A retrospective review of 46 hospitalized patients with laboratory confirmed 2009 H1N1 virus infection was carried out.

Of the 46 patients studied, 20 (43%) were admitted to the ICU and 7 (15%) died. The mean age of patients was 36.9 ± 12.92 years (range: 15-66, median: 32) and only one patient was over 65 years old. The male-to-female ratio was 1.3:1 (26:20). Out the total of 46, 7 patients (15.2%) were cigarette smokers and 10 (21.7%) were drug abusers (Including; opium inhalation in eight patients and intravenous drug abuse (IVDA) in two of them). History of drug abuse was more common among ICU admitted patients and the opium inhalation rate was significantly common in these patients (*P* = 0.01) [Table 1].

**Table 1:** Baseline characteristics of hospitalized patients with laboratory-confirmed 2009 pandemic (H1N1) influenza

Characteristic	Total (*n* = 46)	ICU (*n* = 20)	Hospital (other than ICU) (*n* = 26)	*P* value*
Demographic factors				
Age in years				
Mean	35.91 ± 1.3	36.9 ± 12.9	35.15 ± 13.17	0.655¶
Range	15-66	21-66	15-61	
Female gender, %	43.5	50	38.4	0.31†
Smoker, %	15.2	20	11.5	0.35‡
Opium use, %	17.4	35	3.8	0.01‡
IVDU, %	4.3	10	0	0.18‡
Alcohol use, %	0	–	–	
Close contact with H1N1 cases, %	10.9	5	15.4	0.36‡
Mean time interval between beginning of symptoms to admission, days	5.6 ± 3.8	6.4 ± 3.5	5 ± 3.9	
Reported symptoms, %#				
Cough	97.8	95	100	0.43‡
Fever	91.3	90	96.2	0.57‡
Chills	80.4	85	80.8	0.99‡
Sputum	78.3	95	65.4	0.02‡
Dyspnea	78.3	90	69	0.08‡
Myalgia	69.6	70	69.2	0.95‡
Chest pain	39.1	55	26.9	0.05‡
Sweat	37	50	26.9	0.10†
Diarrhea	34.8	35	34.6	0.97†
Hemoptysis	30.4	55	11.5	0.03‡
Vomiting	28.3	20	34.6	0.33‡
Headache	15.2	5	23.1	0.11‡
Altered mental status	13	30	0	0.004‡
Abdominal pain	8.7	5	11.5	0.62‡
Loss of consciousness	8.7	20	0	0.03‡
Sore throat	4.3	0	7.7	0.49‡
Coinfection with seasonal influenza A	32.6	30	34.6	0.49†
Outcome, %				
Need to mechanical ventilation	26.1	60	0	0.001‡
ICU stay, day	N/A	14.63 ± 13.11	N/A	N/A
Ventilation day	N/A	6.26 ± 11.48	N/A	N/A
Death	15.2	30	3.8	0.03‡

* *P* values are for the comparison of patients admitted to ICU and regular wards; missing data were excluded.

¶ The *P* value was calculated with the use of the *t*-test.

† The *P* value was calculated with the use of a two-sided χ^2^-tsest.

‡ The *P*value was calculated with the use of a two-sided Fisher’s exact test because of the small number of patients (in one or both groups)

# Patients could have more than one symptom; ICU: Intensive care unit, IVDU: Intra venous drug user

Of the 46 patients, 21 (45.7%) had at least one underlying medical condition and the most common coexisting condition was asthma with the incidence of 8 (17.4 %) [Table 2].

**Table 2:** Co-existing conditions, para clinical data, and treatment regimen of hospitalized patients with laboratoryconfirmed 2009 pandemic (H1N1) influenza

Characteristic	Total (*n* = 46)	ICU (*n* = 20)	Hospital (other than ICU) (*n* = 26)	*P* value*
Lab data, %				
Leukocytosis	25.6	25	26.1	0.9^†^
Leukocytopenia	11.6	20	4.3	0.1^‡^
Thrombocytopenia	41.9	55	30.4	0.1^†^
Anemia	34.9	35	34.8	0.9^‡^
Elevated LFT	65.2	85	54.2	0.05^‡^
CPK	440 ± 567	639 ± 696	259 ± 342	0.02^║^
LDH	825 ± 609	115±727	550 ± 286	0.001^║^
ESR	45.9 ± 35.2	47.5 ± 36.4	44.3 ± 35	0.7^║^
O_2_ saturation	79.3 ± 12.9	70 ± 12.2	87.3 ± 6.8	0.000^║^
Cr	1.1 ± 1.1	1.4 ± 1.57	0.9 ± 0.2	0.16^║^
Positive sputum culture for bacteria, %	19.6	30	11.5	0.1^‡^
Acinetobacter	8.7			
Pseudomonas	6.5			
Coagulase negative staphylococci	4.3			
Positive blood culture, %	10.9	15	7.7	0.6^‡^
* E. coli*	4.3			
Pseudomonas	4.3			
Acinetobacter	2.3			
Abnormal chest X-ray findings, %	73.9	95	57.7	0.006^‡^
Bilateral	80	90	66.7	0.1^†^
Ground glass	42.9	50	33.3	
Patchy	32.6	30	33.3	
Consolidation	25.7	20	33.3	
Pleural effusion	8.7	20	0	0.03^‡^
Pneumothorax	4.3	10	0	0.2^‡^
Coexisting conditions, %^§^				
Any	45.7	45	46	0.5^†^
Asthma	17.4	20	15	0.7^‡^
Malignancy^¶^	8.7	5	11	0.4^‡^
Hypertension	8.7	5	11	0.4^‡^
Transplantation^‡‡^	4.3	0	8	0.3^‡^
COPD	4.3	5	3.8	0.6^‡^
HIV	2.3	5	0	0.4^‡^
Diabetes	2.3	0	3.8	0.5^‡^
Chronic renal failure	2.3	5	0	0.4^‡^
Primary immune deficiency^§§^	2.3	5	0	0.4^‡^
Treatment regimen				
Oseltamivir	100	100	100	
Antibiotic	97.8	100	97.8	0.5^‡^
Ceftriaxone	93.5	100	88.5	0.2^‡^
Azithromycin	95.7	100	92.3	0.4^‡^
Vancomycin	52.2	90	23.1	0.000^‡^
Corticosteroids	73.9	95	57.7	0.006^‡^
Intravenous immunoglobulin	4.3	10	0	0.1^‡^

The clinical signs and symptoms included productive cough, hemoptysis, chest pain, confusion, and loss of consciousness (*P* < 0.05) [Table 1].

Blood culture and sputum culture were performed for all admitted patients. Positive sputum and/or blood culture was more common in ICU admitted patients. All patients underwent chest radiography on admission and 74% (34/46) had abnormal chest radiograph. Bilateral alveolar opacity was the most common pattern (32.6%). The following findings were relevant to ICU patients: pleural effusion (*P* = 0.006), laboratory test results indicated higher prevalence of leukopenia, thrombocytopenia and increased level of Cr, elevated liver enzymes, CPK and LDH level (*P* < 0.05), low O_2_ saturation level (*P* < 0.001) [Table 2].

In patients who died, antiviral treatment was initiated at a mean time of 7.42 ± 4.07 days after the onset of illness which was longer than that of the ICU patients (6.42 ± 3.54) and other patients who survived (5.29 ± 3.68). Mean ICU stay duration was longer among patients who died (21.71 ± 19.05 vs. 10.5 ± 5.7, *P* = 0.07). Mortality findings included: elderly patients (mean age: 43 ± 14.29 vs. 34.64 ± 12.46), higher levels of ESR, and long-term mechanical ventilation (mean ventilation day: 15.14 vs. 1.08, *P* = 0.006).

Out of the total number of 46, 15 were co-infected with seasonal influenza A virus, including 2 out of 7 of dead patients (*P* > 0.05). Six out of 15 co-infected patients needed ICU admission during their hospitalization. To analyze for the ICU admission and mortality, a multivariate logistic regression analysis was performed and revealed no significant difference between study groups.

## Discussion

During the spring of 2009, pandemic H1N1 influenza virus caused human infection and acute respiratory illness in Mexico.[3] Rates of hospitalization and death have varied widely according to country.[10] In the United States, approximately 9-31% of hospitalized patients with pandemic influenza were admitted to the ICU, with a mortality rate of 14-46%.[11–14] As there were limited data about developing countries, we evaluated factors associated with ICU admission and in-patient mortality in a tertiary care center in Iran.

Although some underlying conditions are known as risk factors for complications from 2009 H1N1 virus infection,[15] we did not find any significant difference between the ICU patient admissions and other patients. More than half of patients (54%) with 2009 H1N1 virus infections who were hospitalized had no reported coexisting medical conditions. This finding is compatible with other studies around the globe.[11,13,14,16] Only one survived ICU patient was HIV-1 positive. The principal clinical symptoms leading to ICU admission were chest pain, hemoptysis, purulent sputum, altered mental status, loss of consciousness, and severe hypoxemia. These findings are similar to the finding of other studies.[13,14]

Like previous studies[3,12,14] laboratory findings include normal or low leukocyte counts. Elevations in levels of serum aminotransferases, LDH, CPK, Cr, and ESR were documented in ICU patients. The presence of thrombocytopenia was a common finding, but there was no significant difference between the two groups.

Radiographic findings in chest X-ray commonly included bilateral ground-glass opacities, patchy infiltrates, and alveolar consolidation. Among various radiographic findings, pleural effusions were considered as an ICU admission risk factor.

Bacterial pneumonia was not a common finding in either patient population. Although usually it is caused by *Staphylococcus aureus* (often methicillin-resistant), Streptococcus pneumoniae, and S. *pyogenes*,[13,14,17] our findings indicated Acinetobacter, Pseudomonas, and Coagulase negative staphylococci in a minority of patients.

The burden and nature of the disease in developing countries is still incompletely understood.[18] The study has some limitations, including retrospective design, small study population, and being conducted in a tertiary referral center. Due to limitations listed above, some important information, like the history of seasonal flu vaccination, was missing.

The mortality rate in the study population was high but compared favorably with other published studies. Severe pandemic H1N1 influenza necessitating admission to the ICU was associated with delayed initiation of antiviral therapy, history of opium inhalation, and some symptoms including; productive cough, hemoptysis, chest pain, confusion, and loss of consciousness.

A significant higher rate of opium addiction in ICU patients supports the idea that it could predispose patients to severe infection [Table 1]. So this risk factor could be more evaluated during the future pandemics.

As Frederick *et al*. believe delay in antiretroviral therapy is a risk factor for severe infection and mortality, the results confirmed that the idea, however, it was not significant due to small number of population study (7.42 ± 4.07 vs. 5.29 ± 3.68).[15]

Further investigation of respiratory specimens revealed 32.6% of patients had coinfection of seasonal influenza A and pandemic H1N1 virus. However, this finding has no significant relation with mortality. This finding should be considered during future potential pandemics. Despite these associations, the lack of a control group limits the ability to extrapolate from this observation. Further research and analysis is required for clarification of many complex and interrelated factors involved in the outcomes of pandemic HINI influenza.
